# A new discriminant strategy combined with four TIRADS screening procedures increases ultrasound diagnostic accuracy—focusing on “wrong diagnostic” thyroid nodules

**DOI:** 10.1007/s00330-022-09126-2

**Published:** 2022-09-28

**Authors:** Ke Lu, Long Wang, Shuiqing Lai, Zhijiang Chen, Shuzhen Cong, Chunwang Huang, Kehong Gan, Haixia Guan, Jian Kuang

**Affiliations:** 1 Department of Endocrinology, First Affiliated Hospital of Guangzhou Medical University, Guangzhou, Guangdong China; 2 Department of Endocrinology, Guangdong Provincial People’s Hospital, Guangdong Academy of Medical Sciences, Guangzhou, Guangdong China; 3 The Second School of Clinical Medicine, Southern Medical University, Guangzhou, Guangdong China; 4 Department of Ultrasound, Guangdong Provincial People’s Hospital, Guangdong Academy of Medical Sciences, Guangzhou, Guangdong China

**Keywords:** Thyroid nodules, Ultrasound, TIRADS, Accuracy

## Abstract

**Objective:**

To utilize the discrepancies of different TIRADS, including ACR-TIRADS, Kwak-TIRADS, C-TIRADS, and EU-TIRADS, to explore methods for improving ultrasound diagnostic accuracy.

**Methods:**

In total, 795 nodules with cytological or surgical pathology were included. All nodules were screened by the four TIRADS according to their diagnostic concordance (Screening procedures, SP). Discriminant strategy (DS) derived from predictor variables was combined with SP to construct the evaluation method (SP+DS). The diagnostic performance of the SP+DS method alone and its derivational methods and two-TIRADS combined tests was evaluated.

**Results:**

A total of 86.8% (269/310) malignant nodules and 93.6% (365/390) benign cases diagnosed by the four TIRADS simultaneously were pathologically confirmed, while 12.0% (95/795) nodules could not be consistently diagnosed by them. The criteria of DS were that iso- or hyper-echogenicity nodules should be considered benign, while hypo- or marked hypo-echogenicity nodules malignant. For 95 inconsistently diagnosed nodules screened by at least two TIRADS, DS performed best with an accuracy of 79.0%, followed by Kwak-TIRADS (72.6%). In the overall sample, the sensitivity and AUC were highest for the SP+DS method compared to the four TIRADS (91.3%, 0.895). Combining ACR-TIRADS and Kwak-TIRADS via parallel test resulted in significant improvements in the sensitivity and AUC compared to ACR-TIRADS (89.2% vs. 81.4%, 0.889 vs. 0.863). Combining C-TIRADS and DS in serial resulted in the highest AUC (0.887), followed by Kwak-TIRADS (0.884), while EU-TIRADS was the lowest (0.879).

**Conclusions:**

For undetermined or suspected thyroid nodules, two-TIRADS combined tests can be used to improve diagnostic accuracy. Otherwise, considering the inconsistent diagnosis of two TIRADS may require attention to the echo characteristics to differentiate between benign and malignant nodules.

**Key Points:**

• *The discrepancies in the diagnostic performance of different TIRADS arise from their performance on inconsistently diagnosed nodules*.

• *ACR-TIRADS improves sensitivity via combining with Kwak-TIRADS in parallel (from 81.4 to 89.2%), while C-TIRADS increases specificity via combining with EU-TIRADS in serial (from 80.9 to 85.7%)*.

• *If the diagnostic findings of two TIRADS are inconsistent, echo characteristics will be helpful for the differentiation of benign and malignant nodules with an accuracy of 79.0%*.

**Supplementary Information:**

The online version contains supplementary material available at 10.1007/s00330-022-09126-2.

## Introduction

Ultrasonography, as a simple, non-invasive diagnostic method, now occupies a priority position in the thyroid nodule evaluation process [1]. Certain ultrasound indices are significantly associated with thyroid cancer. Commonly used real-time ultrasound indices include size, composition, shape, halo sign, echogenicity, calcification, and some accessory features, including extrathyroidal extension, lymph nodes, blood flow, and elasticity. In fact, the sensitivity and specificity of any single ultrasound feature for diagnosing thyroid cancer are difficult to reach more than 90% simultaneously. Hypoechoic and solid nodules have higher diagnostic sensitivity but lower specificity, while nodules with microcalcifications, infiltrative margins, and taller-than-wide shapes have higher specificity but lower sensitivity [2]. Therefore, an ultrasound model consisting of a combination of valid ultrasound features is more helpful for identifying the nature of nodules.

In 2009, Chilean scholars first introduced the concept of TIRADS and defined ten ultrasound patterns to distinguish benign and malignant thyroid nodules [3]. Kwak then proposed a simplified stratified assessment system containing only five ultrasound indices including shape, echogenicity, structure, calcification, and margin in 2011 [4]. Subsequently published TIRADS, including ATA guidelines, EU-TIRADS, ACR-TIRADS, and KTA/KSThR-TIRADS, also have been constructed based on these five ultrasound modes. These TIRADS are currently clinically validated and have good diagnostic value. But the definitions of some features within their ultrasound lexicons (e.g., hypoechoic, solid, spongiform) are currently not uniform. And the number of assessment classifications, specific malignant features involved, and even the ways in utilizing suspicious ultrasonic features vary (i.e., calculating the number of suspicious features or using ultrasonic pattern for risk stratification), which make the malignancy rate of the classification from low suspicion to high suspicion different among these systems [5–8].

There is no perfect TIRADS to date. Various TIRADS have their own advantages. For example, the EU-TIRADS and ATA guidelines are pattern-dependent systems characterized by a high negative predictive value and sensitivity, whereas the ACR-TIRADS is a typical score-based system with a high positive predictive value and specificity [9–11]. We assume that various TIRADS will probably form complementary relationships based on these facts. For example, some TIRADS are more applicable in some thyroid nodular cases, while other TIRADS cannot classify them correctly. Further, is it possible to explore new methods to improve the diagnostic accuracy based on data from those unmatched nodules?

Thus, in this study, we focused on the differences of unmatched findings among four TIRADS (including the newly released C-TIRADS) [12]. We then explored potential ways such as two-TIRADS parallel or serial tests or one TIRADS combined with specific ultrasound features to improve the diagnostic accuracy.

## Methods

This retrospective study was approved by the Institutional Review Board, and the requirement for informed consent to review images and medical records was waived.

### Patients

**Fig. 1:**
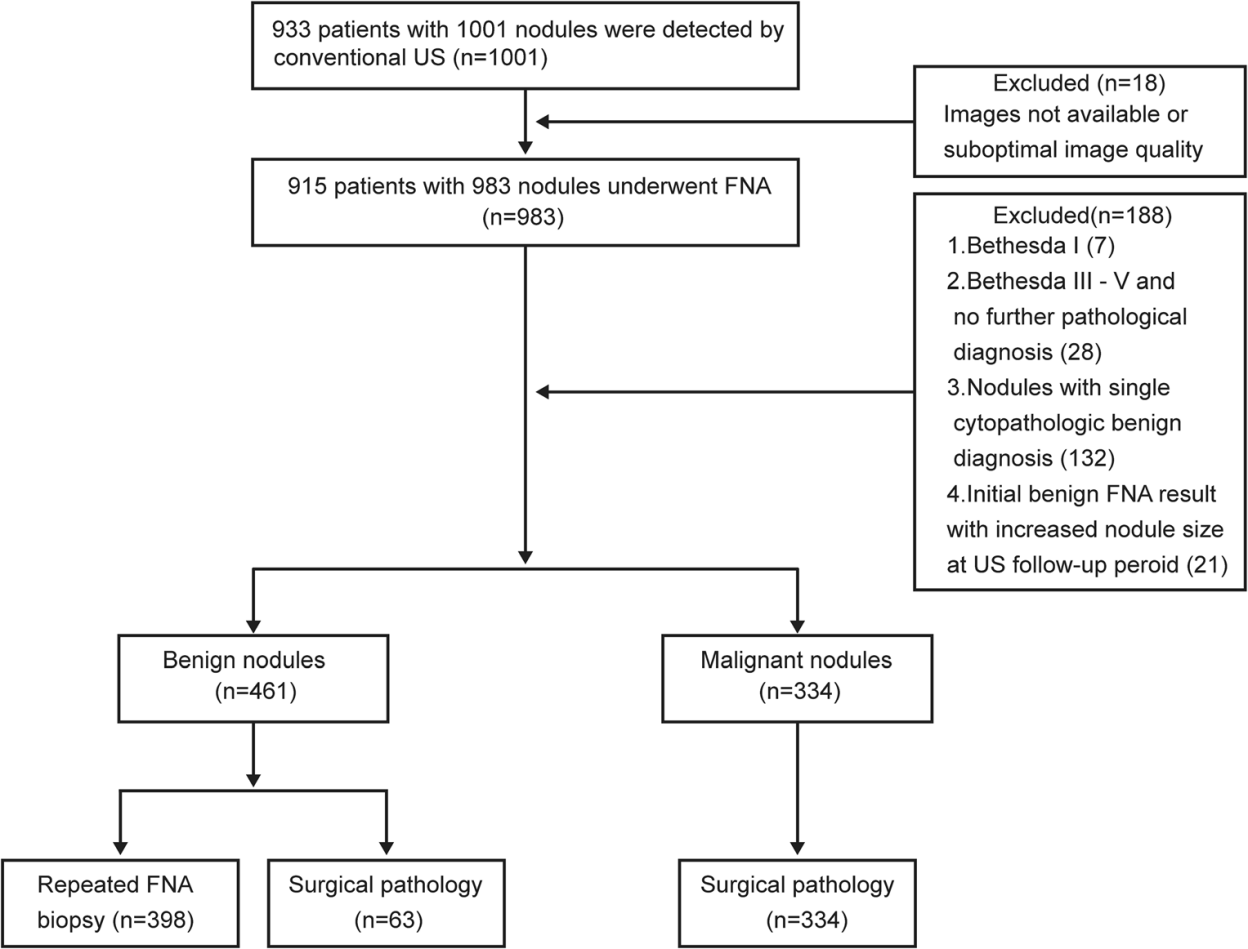
Study flowchart

From February 2016 to February 2019, 1001 thyroid nodules in 933 patients were enrolled in the study. Only definitely diagnosed nodules were included, malignant nodules were confirmed by surgical pathology, and benign nodules were diagnosed by surgical pathology or repeated Bethesda II findings. Based on the above criteria, 795 nodules were finally included, which involved 334 surgical malignant nodules, 63 benign surgical nodules and 398 nodules with repeated Bethesda II results. One hundred eighty-eight nodules that could not be clearly diagnosed were excluded: 7 nodules with Bethesda I cytopathology and 28 Bethesda III-V nodules with no further surgical pathology, 132 nodules with a single benign cytopathologic result, and 21 nodules with initial benign pathology but with increased nodular size in the follow-up period by ultrasound examination (mean interval of 21 months, range 2 to 35 months) (Fig. 1).

### Sonography examination and image evaluation

Conventional ultrasound examinations were performed using Aplio 500 (Toshiba Medical System), HI Vision Ascendus (Hitachi Medical Corporation), or HI Vision Preirus (Hitachi Medical Corporation) ultrasound instruments equipped with 5–12-MHz linear array transducers by board-certified radiologists. The ultrasound images were reviewed by one radiologist with more than 20 years of experience in thyroid ultrasound diagnosis and recorded by two experienced endocrinologists with the help of the radiologist. They were all blinded to the patients’ fine-needle aspiration (FNA) results or pathological diagnosis before sonography examination. In case of disagreement, conclusions would be drawn by consensus. Before assessing nodules, we studied and compared the lexicon and classification of four TIRADS (Supplementary Tables 1 and 2). The definition and classification of the various TIRADS regarding composition, echogenicity, margin, shape, and calcification are substantially similar. However, there are slight differences in the definition of solid, spongiform, hypoechoic, and section to evaluate the nodular orientation. C-TIRADS and Kwak-TIRADS are both counting-based systems. C-TIRADS included marked hypo-echogenicity and ill-defined margin into the scoring system and considered the presence of comet tail artifacts as a minus item.

### FNA, cytopathology, and histopathology

Thyroid nodules were judged as benign or malignant according to FNA cytopathology or surgical histopathology. The surgical pathological diagnosis was based on the WHO diagnostic criteria [13], and the cytopathological classification was based on the Bethesda system of thyroid FNA cytology proposed by the National Cancer Institute [14]. Informed consent was obtained from all patients before the FNA biopsy. The procedure was performed by an endocrinologist experienced in puncture using ultrasound-guided FNA technique by a color doppler ultrasound scanner with an L14-5 high-frequency line array probe (Ultrasonix Medical Ltd., Sonix SP). Benign pathology was defined by repeated Bethesda II results according to the recommendation by the guidelines about ablation treatment [1, 15]. At our institution, the requirement of repeated FNAs meets the following situations: (1) The puncture results are Bethesda I, III, and IV, requiring repeated confirmation or performing further genetic test; (2) Nodules are categorized as intermediate or high suspicion according to TIRADS assessment, but the puncture results are Bethesda II; (3) Patients are scheduled to undergo thermal ablation treatment; (4) During the follow-up period, nodules are with a rapid increase in diameter or volume, or development of new suspicious features including margin, echogenicity, calcification, etc. The time interval was 2–4 weeks between two repeated benign FNAs.

### Statistical analysis

SPSS 26 software (IBM) and MedCalc 19.0.4 software (MedCalc) were used for statistical analysis. Quantitative data conforming to normal distribution were presented as mean ± standard deviation and evaluated by independent samples t-test. Measurement data that did not conform to a normal distribution were expressed as median and interquartile ranges and evaluated by a nonparametric test. Qualitative data were expressed as frequencies and evaluated by a chi-squared test. The optimal cut-off point of each TIRADS was determined from ROC analysis when the Youden index was the highest, as well as sensitivity and specificity. Sensitivity, specificity, positive predictive value (PPV), negative predictive value (NPV), and area under the curve (AUC) were calculated. The McNemar test was used to assess the differences in parameters. Stepwise discriminant analysis was done to determine variables that may discriminate between benign and malignant nodules. Two-sided *p* values < 0.05 were considered statistically significant.

## Results

### Baseline

There were no significant differences in the age and gender of patients with benign and malignant nodules. The maximum size of malignant nodules was significantly smaller than that of benign nodules (median 1.00 [Q1-Q3, 0.80-1.50] vs. 2.40 [Q1-Q3, 1.50-3.20]) (Supplementary Table 3). The number of surgical pathological papillary thyroid carcinoma (PTC) was 334. There were 52 surgical pathologies of nodular goiter, three adenomatous goiters, six follicular thyroid adenoma, and two nodular Hashimoto thyroiditis among benign nodules.

As shown in Table 1, the malignant rate of ACR-TIRADS TR3 and TR4, C-TIRADS CTR 4b, and EU-TIRADS grade 4 were higher than the recommended malignancy rate. There were significant differences between the four TIRADS grades (all *p* < 0.001).

**Table 1:** Estimated malignant risk of the four TIRADS according to pathological diagnosis

			Pathological diagnosis		Recommended	Calculated	*p*
		Total	Benign	Malignancy	Malignant	Malignant	
		*N* = 795	*N* = 461	*N* = 334	risk	risk	
ACR-TIRADS							< 0.001
	TR2	127 (15.9%)	127 (27.5%)	0 (0%)	≤ 2%	0%	
	TR3	201 (25.3%)	190 (41.2%)	11 (3.3%)	< 5%	5.5%	
	TR4	154 (19.4%)	103 (22.3%)	51 (15.3%)	5–20%	33.1%	
	TR5	313 (39.4%)	41 (9.0%)	272 (81.4%)	> 20%	86.9%	
Kwak-TIRADS							< 0.001
	3	141 (17.7%)	139 (30.1%)	2 (0.6%)	2.0–2.8%	1.4%	
	4a	226 (28.4%)	215 (46.6%)	11 (3.3%)	3.6–12.7%	4.9%	
	4b	80 (10.1%)	54 (11.7%)	26 (7.7%)	6.8–37.8%	32.5%	
	4c	276 (34.7%)	48 (10.5%)	228 (68.3%)	21–91.9%	82.6%	
	5	72 (9.1%)	5 (1.1%)	67 (20.1%)	88.7–97.9%	93.1%	
C-TIRADS							< 0.001
	CTR2	10 (1.2%)	10 (2.1%)	0 (0%)	0%	0%	
	CTR3	142 (17.8%)	139 (30.1%)	3 (0.9%)	≤ 2%	2.0%	
	CTR4a	249 (31.3%)	224 (48.6%)	25 (7.5%)	2–10%	10.0%	
	CTR4b	124 (15.6%)	58 (12.6%)	66 (19.7%)	10–50%	53.2%	
	CTR4c	257 (32.4%)	29 (6.3%)	228 (68.2%)	50–90%	88.7%	
	CTR5	13 (1.7%)	1 (0.3%)	12 (3.7%)	≥ 90%	92.3%	
EU-TIRADS							< 0.001
	2	7 (0.9%)	7 (1.5%)	0 (0%)	0%	0%	
	3	323 (40.6%)	315 (68.3%)	8 (2.4%)	2–4%	2.5%	
	4	87 (10.9%)	65 (14.1%)	22 (6.6%)	6–17%	25.3%	
	5	378 (47.6%)	74 (16.1%)	304 (91.0%)	26–87%	80.4%	

Supplementary Table 4 shows the diagnostic performance of the four TIRADS. The results showed that the best diagnostic cut-off values of ACR-TRADS, Kwak-TIRADS, C-TIRADS and EU-TIRADS were TR5, 4c, CTR 4b, and grade 5, respectively. C-TIRADS had the highest sensitivity (91.6%) and NPV (93.0%), while ACR-TIRADS had the highest specificity (91.1%) and PPV (86.9%). However, Kwak-TIRADS had the highest AUC 0.884 (95% CI:0.860-0.906).

**Fig. 2:**
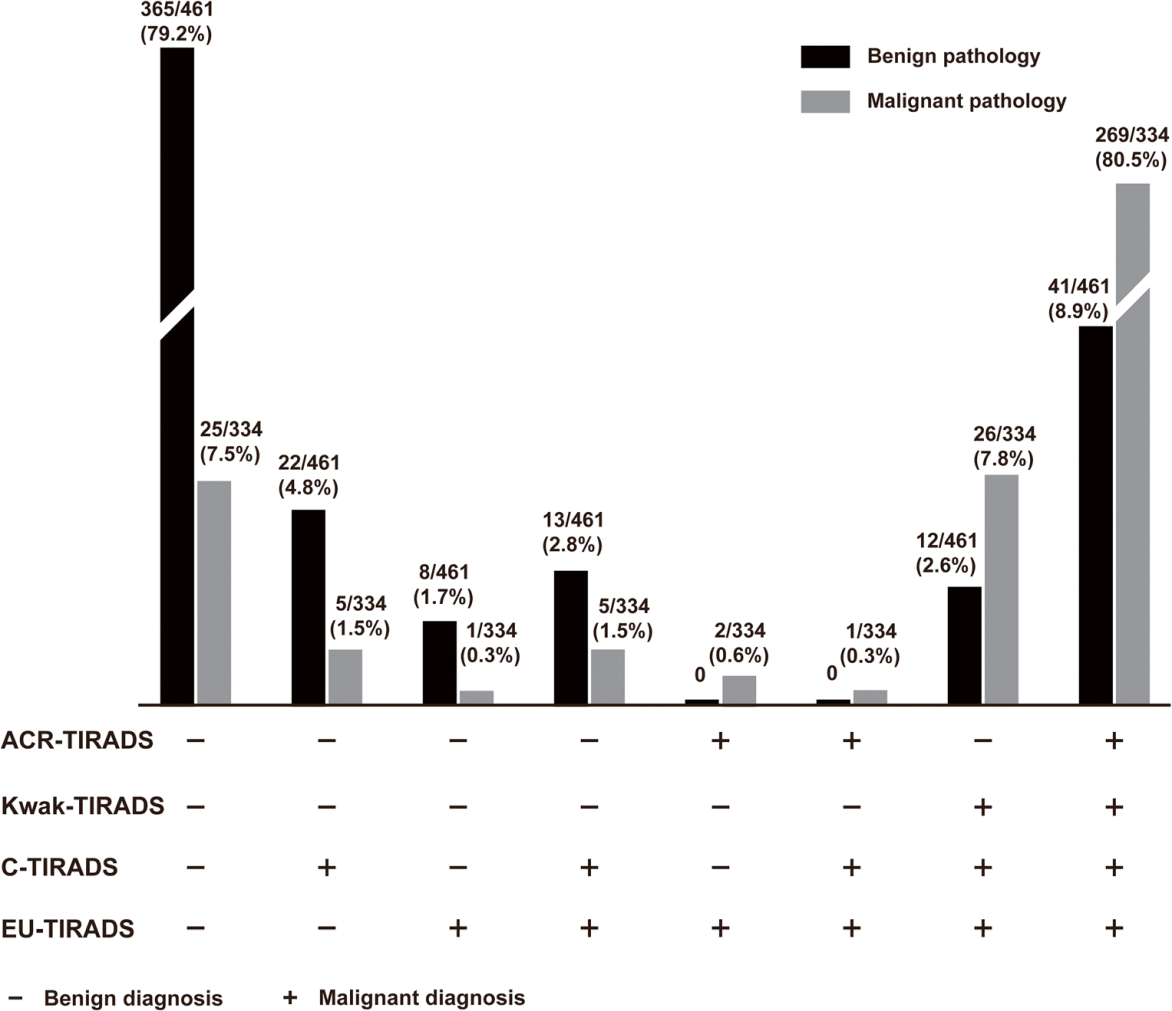
Frequency distribution plot of different TIRADS in assessing pathological benign and malignant nodules. The definitions for the malignant and benign results under optimal cut point are as follows: If we set category 5 of ACR-TIRADS as the best cut-off point value, diagnostic malignant nodules are set to be category 5, whereas category 1 to 4 indicates diagnostic benign nodules; If category 5 of EU-TIRADS is set to be best cut-off point value, category 5 indicates diagnostic malignant nodules, while category 1 to 4 represents diagnostic benign nodules; If we set category 4b of C-TIRADS as best cut-off point value, category 4b or 4c or 5 is set to be diagnostic malignant nodules, whereas category 1 to 4a indicates diagnostic benign nodules. If category 4c of Kwak-TIRADS is set to be the best cut-off point value, category 4c or 5 indicates diagnostic malignant nodules, while category 1 to 4b represents diagnostic benign nodules

Figure 2 illustrates the diagnostic distribution for the four TIRADS in assessing pathological benign and malignant nodules. The number of cases with inconsistent findings of benign pathology was more than that of malignant pathology (96/795 vs. 65/795). In total, 86.8% (269/310) malignant nodules and 93.6% (365/390) benign cases diagnosed by the four TIRADS simultaneously were pathologically confirmed, whereas 8.3% (66/795) of nodules could not be correctly diagnosed by any of the TIRADS, and 12.0% (95/795) nodules could not be consistently diagnosed by all the four TIRADS.

### Discriminant strategy

Most of the 95 nodules were solid, wider-than-tall, without calcifications regardless of pathological diagnosis. Only a small percentage of nodules contained taller-than-wide (1.1%) and microcalcification (8.4%) features. As for echogenic features, benign nodules were predominantly iso/hyperechoic (70.9%), while malignant ones showed predominantly hypoechoic (85.0%). As for margin features, a well-circumscribed margin was predominant in benign nodules (56.4%), and a lobulated or irregular margin was predominant in malignant cases (77.5%) (Supplementary Table 5).

Stepwise discriminant analysis was used to distinguish the variables that best identified benign and malignant nodules. Five commonly used variables were included as predictor variables for malignant thyroid nodules. Stepwise discriminant analysis screened the echogenicity variable (*F* = 34.87, *p* < 0.001). The discriminant function was *Y *= 2.368 × echogenicity−1.421. This discriminant function was statistically different (Wilks’ lambda = 0.74, *p* < 0.001) and had an excellent predictive value as it could correctly predict the classification of 79.0% of cases.

According to the above discriminant function, the discriminant strategy (DS) based on nodular features was as follows: Iso- or hyper-echogenicity nodules should be considered benign. Hypo- or marked hypo-echogenicity nodules should be regarded as malignant. The diagnostic results of this strategy remained consistent with the above prediction results (Table 2).

**Table 2:** Classification results of discriminant analysis and criteria for differentiating nodules subgroups

Actual	Predicted group membership		
	Benign	Malignance	Total
Benign	39 (70.9%)	16 (29.1%)	55 (100%)
Malignance	4 (10.0%)	36 (90.0%)	40 (100%)
Criteria		Echogenicity	
	Iso- or Hyper-	Hypo-	Marked hypo-
	-	+	+

Note. 79.0% of originally inconsistently grouped cases are correctly classified. Plus means a nodule is diagnosed as malignance and minus represents a benign diagnosis based on the different levels of echogenicity. Marked hypo-echogenicity means the echogenicity is lower than that of the strap muscles of the neck

### Performance characteristics

**Fig. 3:**
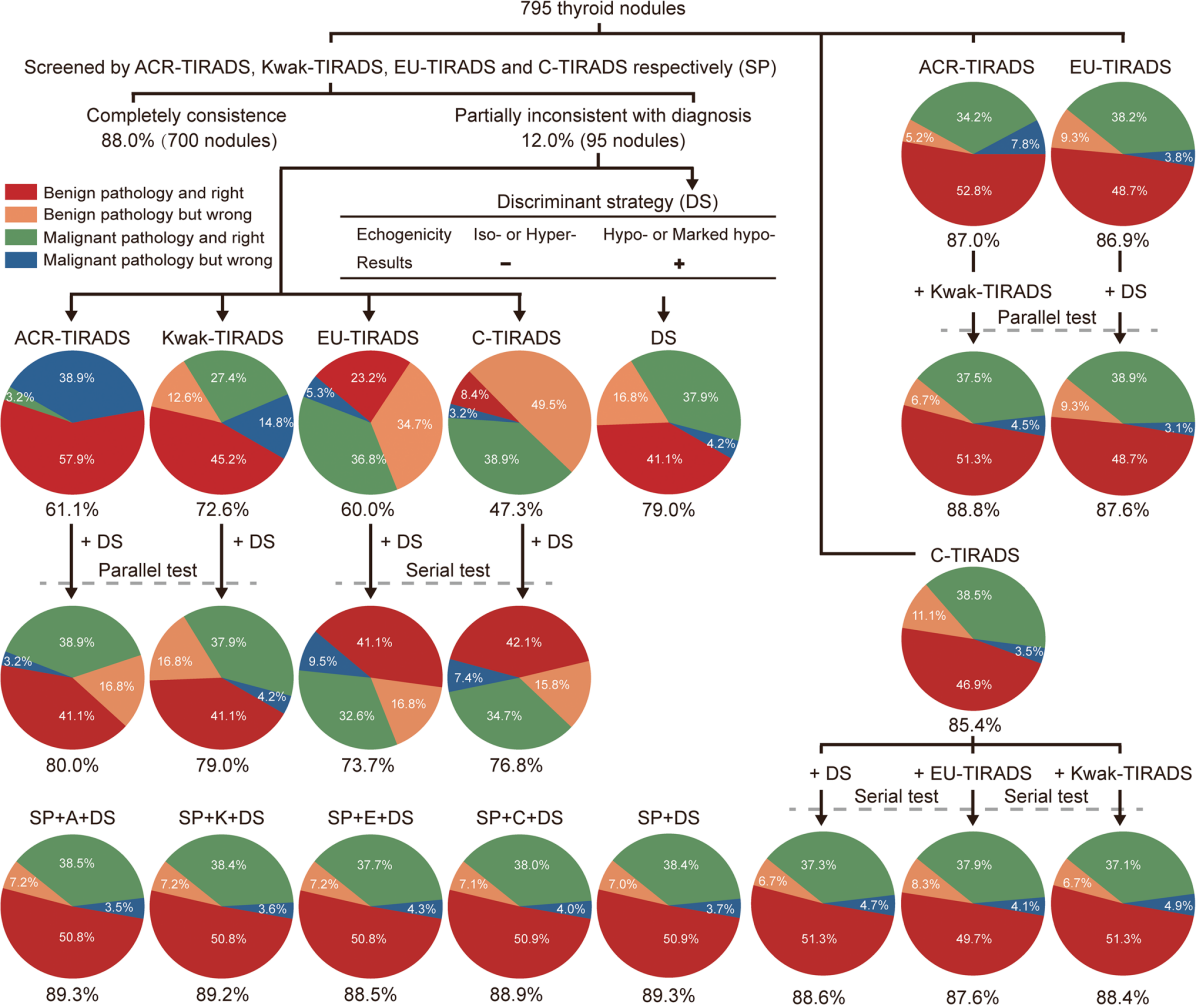
Summary of methods and strategies included in the article analysis process. SP: Screening procedures, DS: Discriminant strategy, SP+DS: The evaluation method consists of the four TIRADS screening procedures with partially inconsistently diagnosed nodules judged by discriminant strategy, SP+A/C/K/E+DS: The evaluation method consists of the four TIRADS screening procedures with partially inconsistently diagnosed nodules judged by ACR-TIRADS/C-TIRADS/Kwak-TIRADS/EU-TIRADS and combined with discriminant strategy. The parallel test is defined as follows: The same nodule is defined as benign only when both tests are diagnosed as benign, or malignance when one of the tests is diagnosed as malignance. The serial test is defined as follows: The same nodule is defined as malignance only when both tests are diagnosed as malignance, or benign when one of tests is diagnosed as benign. The dotted line indicates that the two longitudinal TIRADS or TIRADS and discriminant strategy (DS) are combined in parallel or serial. The numbers at the bottom of the pie chart represent accuracy

For 95 inconsistently diagnosed nodules screened by at least two TIRADS, DS performed best with an accuracy of 79.0%, followed by Kwak-TIRADS (72.6%) (Fig. 3). Table 3 examines the connection modes of various TIRADS and DS for multiple TIRADS inconsistently diagnosed nodules. Combining DS and ACR-TIRADS in parallel resulted in a significant increase in accuracy (from 61.1 to 80.0%), and the AUC of A-DS was significantly improved (0.817 vs. 0.538) compared to those of ACR-TIRADS alone, while a serial test combining DS and C-TIRADS also resulted in a sharp increase of accuracy (from 47.3 to 76.8%), and the AUC of C-DS method was significantly improved (0.776 vs. 0.535) compared to those of C-TIRADS alone. Regardless of using any combined tests, the AUC of combining DS and EU-TIRADS was substantially higher than that of EU-TIRADS alone (0.700 vs. 0.637, 0.742 vs. 0.637). But the serial test may be preferred because of the higher AUC value and the more balanced sensitivity and specificity values.

**Table 3:** Diagnostic performance of different TIRADS combined with discriminant strategy using parallel or serial tests on partially inconsistently diagnosed nodules subgroups

*N* = 95	Sensitivity (%)	Specificity (%)	AUC (95% CI)	*p*
To increase sensitivity (parallel test)				
A-DS	92.5	70.9	0.817 (0.725–0.889)	< 0.001
A	7.5	100.0	0.538 (0.432–0.640)	
E-DS	100.0	40.0	0.700 (0.597–0.790)	0.0183
E	87.5	40.0	0.637 (0.532–0.734)	
C-DS	100.0	12.7	0.564 (0.458–0.665)	0.2160
C	92.5	14.6	0.535 (0.430–0.638)	
K-DS	90.0	70.9	0.805 (0.710–0.879)	0.0227
K	65.0	78.2	0.716 (0.614–0.804)	
To increase specificity (serial test)				
A-DS	5.0	100.0	0.525 (0.420–0.628)	0.3173
A	7.5	100.0	0.538 (0.432–0.640)	
E-DS	77.5	70.9	0.742 (0.642–0.826)	0.0082
E	87.5	40.0	0.637 (0.532–0.734)	
C-DS	82.5	72.7	0.776 (0.679–0.855)	< 0.001
C	92.5	14.6	0.535 (0.430–0.638)	
K-DS	65.0	78.2	0.716 (0.614–0.804)	1.0000
K	65.0	78.2	0.716 (0.614–0.804)	

*A*, ACR-TIRADS; *E*, EU-TIRADS; *C*, C-TIRADS; *K*, Kwak-TIRADS; *DS*, discriminant strategy; *AUC*, area under the curve; *CI*, confidence interval. The parallel test is defined as follows: The same nodule is defined as benign only when both TIRADS and DS are diagnosed as benign, or defined as malignance when one of tests is diagnosed as malignance. The serial test is defined as follows: The same nodule is defined as malignance only when both TIRADS and DS are diagnosed as malignance, or defined as benign when one of tests is diagnosed as benign

Table 4 shows the diagnostic performance of assessment methods built from the screening process, the DS, and combined tests in the overall sample. The sensitivity and AUC were highest for the SP+DS method compared to the four TIRADS (91.3%, 0.895). The specificity was highest for ACR-TIRADS (91.1%), followed by Kwak-TIRADS and the SP+DS method with no significance between them (88.5% vs. 87.6%, *p* > 0.05). When evaluating new methods including combined tests, the sensitivity and AUC were highest for the SP+A+DS method (Parallel mode) (91.6%, 0.896), while the specificity and AUC was highest for the SP+C+DS method (Serial mode) (87.9%, 0.891). For a total of 31 initial Bethesda 3 and 4 nodules (3.9%, 31/795), of which 17 (54.8%) were pathologically malignant and 14 (45.2%) benign, the frequency of correct diagnosis was highest for the SP+DS method and C-TIRADS (both were 20/31), followed by Kwak-TIRADS (19/31) (Supplementary Table 6).

**Table 4:** Diagnostic performance of evaluation methods consisting of the discriminant strategy alone or combined with the four TIRADS using serial or parallel tests after the screening procedures

*N* = 795	Sensitivity (%)	Specificity (%)	AUC (95% CI)
A	81.4 (76.8–85.5) ^a^**	91.1 (88.1–93.5) ^a^**	0.863 (0.837–0.886) ^a^**
K	88.3 (84.4–91.6) ^a^**	88.5 (85.2–91.3)	0.884 (0.860–0.906) ^a^*
C	91.6 (88.1–94.4)	80.9 (77.0–84.4) ^a^**	0.863 (0.837–0.886) ^a^**
E	91.0 (87.4–93.9)	84.0 (80.3–87.2) ^a^**	0.875 (0.850–0.897) ^a^**
SP+DS	91.3 (87.8–94.1)	87.6 (84.3–90.5)	0.895 (0.871–0.915)
Parallel test			
SP+A+DS	91.6 (88.1–94.4) ^b^**	87.6 (84.3–90.5) ^b^**	0.896 (0.873–0.917) ^b^**
SP+K+DS	91.3 (87.8–94.1) ^c^**	87.6 (84.3–90.5)	0.895 (0.871–0.915) ^c^*
Serial test			
SP+C+DS	90.4 (86.7–93.4)	87.9 (84.5–90.7) ^d^**	0.891 (0.868–0.912) ^d^**
SP+E+DS	89.8 (86.1–92.8)	87.6 (84.3–90.5) ^e^**	0.887 (0.863–0.908) ^e^*

*A*, ACR-TIRADS; *E*, EU-TIRADS; *C*, C-TIRADS; *K*, Kwak-TIRADS; *AUC*, area under the curve; *CI*, confidence interval; *SP*, screening procedures; *DS*, discriminant strategy; *SP+DS*, the evaluation method consists of the four TIRADS screening procedures with partially inconsistently diagnosed nodules judged by discriminant strategy; *SP+A/K/C/E+DS*, the evaluation method consists of the four TIRADS screening procedures with partially inconsistently diagnosed nodules judged by ACR-TIRADS/Kwak-TIRADS/C-TIRADS/EU-TIRADS and combined with discriminant strategy. The parallel test is defined as follows: The same nodule is defined as benign only when both TIRADS and DS are diagnosed as benign, or malignance when one of tests is diagnosed as malignance. The serial test is defined as follows: The same nodule is defined as malignance only when both TIRADS and DS are diagnosed as malignance, or benign when one of tests is diagnosed as benign. ^a^ SP+DS is the statistical control group, ^b^ ACR-TIRADS is the statistical control group, ^c^ Kwak-TIRADS is the statistical control group, ^d^ C-TIRADS is the statistical control group, ^e^ EU-TIRADS is the statistical control group. * *p* < 0.05, ** *p* < 0.01

We further examined the performance of one TIRADS combined with another TIRADS or DS in the overall sample (Table 5). Despite a decrease in specificity (from 91.1% to 88.5%), combining ACR-TIRADS and Kwak-TIRADS via parallel test resulted in significant improvements in the sensitivity and AUC compared to ACR-TIRADS (89.2% vs. 81.4%, 0.889 vs. 0.863). Although the p-value is at the boundary for statistical significance for the sensitivity (from 91.0 to 92.5%, *p* = 0.053), combining EU-TIRADS and DS via parallel test resulted in significant improvements in AUC (from 0.875 to 0.882, *p* = 0.0245). There are three ways to improve the specificity of C-TIRADS, including combing with Kwak-TIRADS, EU-TIRADS and DS. Combining C-TIRADS and DS results in the highest AUC (0.887, *p* = 0.0013), followed by Kwak-TIRADS (0.884, *p* = 0.0062), while the lowest AUC was EU-TIRADS (0.879, *p* = 0.0064).

**Table 5:** Diagnostic performance of the discriminant strategy combined with TIRADS or two combined TIRADS using parallel or serial combination strategies

*N* = 795	Sensitivity (%)	Specificity (%)	AUC (95% CI)	Note
A	81.4 (76.8–85.5)	91.1 (88.1–93.5)	0.863 (0.837–0.886)	-
K	88.3 (84.4–91.6)	88.5 (85.2–91.3)	0.884 (0.860–0.906)	-
C	91.6 (88.1–94.4)	80.9 (77.0–84.4)	0.863 (0.837–0.886)	-
E	91.0 (87.4–93.9)	84.0 (80.3–87.2)	0.875 (0.850–0.897)	-
To increase sensitivity (parallel test)				
A-K	89.2 (85.4–92.3)	88.5 (85.2–91.3)	0.889 (0.865–0.910)	Sig
*p*	< 0.001 ^a^	< 0.001 ^a^	0.0016 ^a^	
A-C	92.2 (88.8–94.9)	80.9 (77.0–84.4)	0.866 (0.840–0.889)	NS
A-E	91.0 (87.4–93.9)	84.0 (80.3–87.2)	0.875 (0.850–0.897)	NS
K-C	91.6 (88.1–94.4)	80.9 (77.0–84.4)	0.863 (0.837–0.886)	NS
K-E	91.0 (87.4–93.9)	84.0 (80.3–87.2)	0.875 (0.850–0.897)	NS
E-C	92.5 (89.1–95.1)	79.2 (75.2–82.8)	0.858 (0.832–0.882)	NS
A-DS	95.8 (93.1–97.7)	79.8 (75.9–83.4)	0.878 (0.853–0.900)	NS
K-DS	95.5 (92.7–97.5)	79.6 (75.6–83.2)	0.876 (0.851–0.898)	NS
C-DS	92.5 (89.1–95.1)	80.7 (76.8–84.2)	0.866 (0.840–0.889)	NS
E-DS	92.5 (89.1–95.1)	84.0 (80.3–87.2)	0.882 (0.858–0.897)	Sig
*p*	0.053 ^b^	1.000 ^b^	0.0245 ^b^	
To increase specificity (serial test)				
A-K	80.5 (75.9–84.6)	91.1 (88.1–93.5)	0.858 (0.832–0.882)	NS
A-C	80.8 (76.2–84.9)	91.1 (88.1–93.5)	0.860 (0.834–0.883)	NS
A-E	81.4 (76.8–85.5)	91.1 (88.1–93.5)	0.863 (0.837–0.886)	NS
K-C	88.3 (84.4–91.6)	88.5 (85.2–91.3)	0.884 (0.860–0.906)	Sig
*p*	0.001 ^c^	< 0.001 ^c^	0.0062 ^c^	
K-E	88.3 (84.4–91.6)	88.5 (85.2–91.3)	0.884 (0.860–0.906)	NS
E-C	90.1 (86.4–93.1)	85.7 (82.1–88.8)	0.879 (0.854–0.901)	Sig
*p*	0.064 ^d^	< 0.001 ^d^	0.0064 ^d^	
A-DS	81.1 (76.5–85.2)	91.1 (88.1–93.5)	0.861 (0.835–0.885)	NS
K-DS	88.3 (84.4–91.6)	88.5 (85.2–91.3)	0.884 (0.860–0.906)	NS
C-DS	88.9 (85.1–92.1)	88.5 (85.2–91.3)	0.887 (0.863–0.908)	Sig
*p*	0.004 ^e^	< 0.001 ^e^	0.0013 ^e^	
E-DS	88.3 (84.4–91.6)	88.3 (85.0–91.1)	0.883 (0.859–0.905)	NS

*A*, ACR-TIRADS; *E*, EU-TIRADS; *C*, C-TIRADS; *K*, Kwak-TIRADS; *AUC*, area under the curve; *CI*, confidence interval; *NS*, no significance; *Sig*, Significance. The parallel test is defined as follows: The same nodule is defined as benign only when both tests are diagnosed as benign, or malignance when one of tests is diagnosed as malignance. The serial test is defined as follows: The same nodule is defined as malignance only when both tests are diagnosed as malignance, or benign when one of tests is diagnosed as benign. ^a^ ACR-TIRADS is the statistical control group, ^b^ EU-TIRADS is the statistical control group, ^c, d, e^ C-TIRADS is the statistical control group

**Fig. 4:**
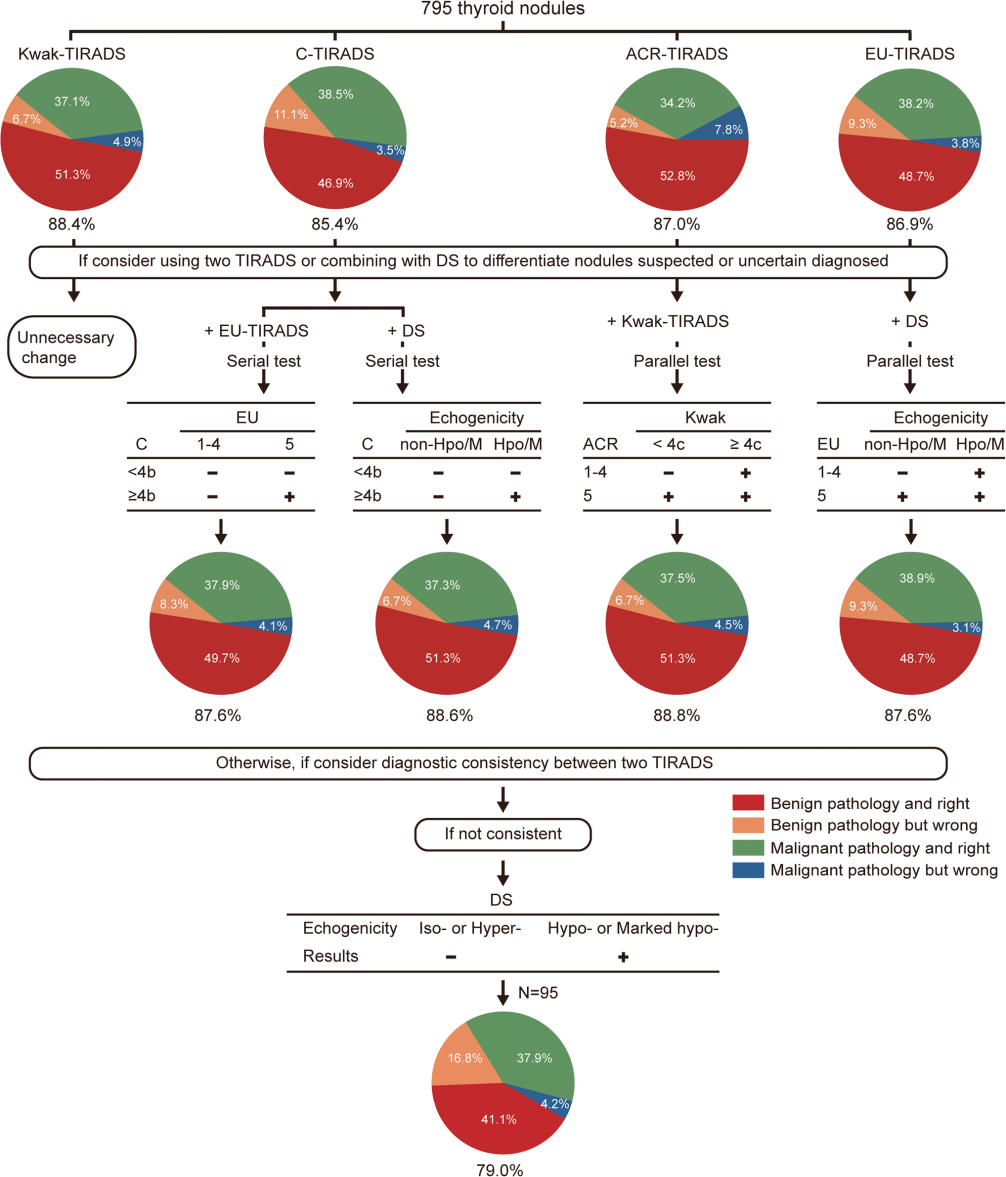
Summary of recommended strategies to improve ultrasound accuracy based on this article’s findings. ACR: ACR-TIRADS, EU: EU-TIRADS, C: C-TIRADS, Kwak: Kwak-TIRADS, DS: Discriminant strategy, Hpo/M: Nodules with hypo- or marked hypo-echogenicity, non-Hpo/M: Nodules with iso- or hyper-echogenicity. The best diagnostic cut-off values of ACR-TRADS, Kwak-TIRADS, C-TIRADS, and EU-TIRADS are TR5, 4c, CTR 4b, and grade 5 in this article. The numbers at the bottom of the pie chart represent accuracy

Figure 4 illustrates recommended strategies to improve ultrasound accuracy based on this article’s findings. For suspicious or indeterminate nodules, it was recommended to use two-TIRADS combined tests or one TIRADS combined with DS. But Kwak-TIRADS could be used alone. If someone considered the inconsistent results of two TIRADS, it was recommended to use the DS directly for judgment.

## Discussion

Our study compared the diagnostic performance of the four TIRADS and showed that all four TIRADS have good diagnostic performance. The four TIRADS screening procedures resulted in 12.0% inconsistently diagnosed nodules. We then tested a strategy focusing on this subgroup of nodules to establish methods for improving diagnostic accuracy. The results showed that established criteria based on the independent variable of echogenicity could fully predict the discriminant results with an accuracy of 79.0%, followed by Kwak-TIRADS (72.6%). The diagnostic performance of the SP+DS method was significantly higher than that of the four TIRADS. Especially, the four TIRADS can substantially improve the diagnostic results of partially inconsistently diagnosed nodules when combined with DS, thus improving the diagnostic performance of the constructed methods (including SP+A+DS, SP+K+DS, SP+E+DS, and SP+C+DS). When the DS was applied to the overall sample, significant improvements in the diagnostic performance of C-TIRADS and EU-TIRADS could be obtained by combining tests. Two-TIRADS parallel or serial tests can also help improve the diagnostic performance of ACR-TIRADS and C-TIRADS by combining Kwak-TIRADS.

In this study, the sensitivity of the four TIRADS ranged from 81.4 to 91.6%, specificity from 80.9 to 91.1%, and AUC 0.863 to 0.884, which indicates that all TIRADS have a good diagnostic performance. C-TIRADS has the highest sensitivity, while ACR-TIRADS has the highest specificity, consistent with the results of previous studies [16–18]. The classification screening results corroborated our hypothesis that the partially inconsistently diagnosed results of the four TIRADS for some nodules are precisely the reason for their differential diagnostic performance.

Without adding other new indicators, we used discriminant analysis to screen out a predictor- echo characteristics. The SP+DS method constructed achieved better diagnostic performance. One could argue whether these malignant indicators depend on the probability distribution of the sample. It must be noted that the remaining nodules partially inconsistent with the diagnosis screened by the four TIRADS are less likely to show highly malignant features such as taller-than-wide, microcalcifications, and infiltrative margins. On the contrary, most of them show less highly malignant features such as solid, hypo-echogenicity, irregular margin and macrocalcifications. Moreover, it can be observed that nodules with highly malignant features often have multiple malignant features simultaneously and are more likely to be correctly diagnosed by various TIRADS [4, 6, 7, 12].

The new evaluation methods have their advantages. The consistent diagnosis of the four TIRADS can provide immediate feedback to increase confidence in confirming the diagnosis. What is more, the false-positive or false-negative rate could be effectively controlled and balanced, reducing the rate of unnecessary punctures and improving diagnostic accuracy, which is currently two essential goals in nodular diagnosis [19]. In addition, the DS has been simplified and easy to master. Considering the ease of use, it is recommended that in practice the four TIRADS screening procedures are best carried out with the help of structured forms or designed procedures. Most importantly, the diagnostic performance of the SP+DS method and other SP-based methods have been improved compared with the four TIRADS. As for the Bethesda 3 and 4 nodules, the correct diagnostic frequency of the SP+DS method was even the highest. However, due to the limited sample size, the latter conclusion must be confirmed in future studies.

We further explored the clinical applicability of the DS and extended it to the overall sample. The results showed that combined modes between the DS and the four TIRADS differed in the partially inconsistently diagnosed nodules. Those TIRADS with high sensitivity, such as EU-TIRADS and C-TIRADS, are applicable to the serial test to improve specificity, while ACR-TIRADS and Kwak-TIRADS with better specificity performance are suitable for the parallel test. These results suggest that the way the DS is applied depends on the different characteristics of each TIRADS [19, 20]. The situation seems to be somewhat different in the overall sample. For example, EU-TIRADS with high sensitivity seemed to be able to continue to improve sensitivity through the parallel test. It should be noted that various kinds of TIRADS combine the DS in different ways, which may also reflect differences in the weighting of echo characteristics in the various TIRADS. For C-TIRADS, both in partially inconsistently diagnosed nodules and in the sample overall, C-TIRADS and DS are combined using a serial test to improve specificity, which may be attributed to the fact that hypo-echogenicity is not a highly malignant risk feature in its lexicon and many nodules diagnosed as malignance by C-TIRADS with ill-defined margin were correctly diagnosed as benign according to the serial strategy [12, 17].

Notably, in our study, Kwak-TIRADS has a good balance in terms of sensitivity and specificity, which is consistent with previous studies [20, 21]. For partially inconsistently diagnosed nodules, Kwak-TIRADS also exhibited the best performance besides the SP+DS method. In the overall sample, ACR-TIRADS in parallel combined with Kwak-TIRADS reduced the false negative rate, and C-TIRADS in serial combined with Kwak-TIRADS reduced the false positive rate. However, although C-TIRADS can be combined with Kwak-TIRADS to improve specificity, the accuracy is the same as Kwak-TIRADS (both 88.4%), so it is recommended to use Kwak-TIRADS directly. Taken together, it may not be necessary to combine another strategy to achieve better diagnostic performance for Kwak-TIRADS.

In the clinical setting, two or more evaluation systems are usually considered for suspicious or indeterminate nodules with few highly malignant features [22]. Considering that the customary use of TIRADS may differ among institutions or individuals, diagnostic combinations that are both accurate and clinically significant need to be examined. Based on the results of this study, if a nodule is suspicious or uncertain diagnosis, two TIRADS or the combination of one TIRADS with DS using a parallel or serial tests can be considered to help improve the accuracy, where Kwak-TIRADS can be directly used without the combination test. On the other hand, the diagnostic consistency of the two TIRADS at the optimal cut point can be examined. If the results of the two selected TIRADS are inconsistent, considering the time cost of the screening process, it is suggested to directly use the DS, which can significantly improve the accuracy.

ACR-TIRADS is a commonly used TIRADS with high specificity, effectively reducing unnecessary FNA rates [17, 18, 23]. But false negatives are a concern. According to the results of this study, we may suggest using a parallel test combined with Kwak-TIRADS for judgment to obtain a balance of sensitivity and specificity. However, as with ACR-TIRADS, there seems to be value in the uneven diagnostic performance of TIRADS [19]. Despite using the serial strategy, some new methods’ specificity does not seem to exceed that of ACR-TIRADS. On the contrary, with the combining strategy, this study has obtained multiple sets of assessment methods with a balance of sensitivity and specificity, even some methods that can enhance sensitivity, e.g., A-DS and K-DS. Whether these methods have clinical application need to be further investigated.

Our study has some limitations. First, all patients in this study with malignant thyroid tumors were confined to PTC. Whether the conclusion of this study applies to other thyroid malignant tumors needs to be verified. Second, the selection of optimal cut-off points for TIRADS, especially considering the balance of sensitivity and specificity, might affect the results of this study. However, the cut-off point of each TIRADS is relatively stable. The ACR-TIRADS and EU-TIRADS are mostly set at category 4 or 5, while the Kwak-TIRADS is set chiefly at category 4c to maximize the balance of sensitivity and specificity to ensure the accuracy of diagnosis [19, 24–26]. As mentioned above, the screening procedures almost exclude nodules with multiple highly malignant features, so it can be predicted that, even though samples might be different, similar criteria may still be obtained according to this research strategy. Further research with larger samples or the other thyroid carcinoma is needed to confirm the above hypothesis. In addition, it must be acknowledged that classification below the optimal cut point also has the risk of malignancy, and it is still necessary to consider whether to carry out an FNA examination based on the size of nodules, personal or family history of cancer, and changes in nodules during the follow-up period. Third, as mentioned in the “FNA, cytopathology and histopathology” section, repeated FNAs are not routinely performed in our institution, which may cause selection bias. What is more, there is still a 1–2% false-negative rate based on repeated results, which might overestimate benign nodules and affect the diagnostic performance of each TIRADS. Further study could use benign surgical pathology results to exclude this potential bias [1, 15, 27].

In conclusion, it is undeniable that various TIRADS have good diagnostic performance, but how to further improve the diagnostic accuracy is a question worth exploring. This study is the first to analyze and compare in detail the misdiagnosed and missed cases of different TIRADS. We explored new methods without additional diagnostic indicators and achieved an effective improvement in accuracy. The recommended strategies our findings provide may help to improve the diagnostic accuracy of ultrasound uncertain or suspicious nodules.

## Supplementary Information

Below is the link to the electronic supplementary material.

Supplementary file1 (DOCX 84.6 KB)

## Abbreviations

ACR-TIRADS: American College of Radiology Thyroid Imaging Reporting and Data System
C-TIRADS 2020: Chinese Guidelines for Ultrasound Malignancy Risk Stratification of Thyroid Nodules
DS: Discriminant strategy
EU-TIRADS: European Thyroid Association Guidelines for Ultrasound Malignancy Risk Stratification of Thyroid Nodules in Adults
FNA: Fine-needle aspiration
Kwak-TIRADS: Kwak Thyroid Imaging Reporting and Data System
NPV: Negative predictive value
PPV: Positive predictive value
PTC: Papillary thyroid carcinoma
SP: Screening procedures
SP+A/C/K/E+DS: The evaluation method consists of the four TIRADS screening procedures with partially inconsistently diagnosed nodules judged by ACR-TIRADS/C-TIRADS/Kwak-TIRADS/EU-TIRADS and combined with discriminant strategy
SP+DS: The evaluation method consists of the four TIRADS screening procedures with partially inconsistently diagnosed nodules judged by discriminant strategy

## Declarations

### Guarantor

The scientific guarantor of this publication is Jian Kuang.

### Statistics and biometry

One of the authors (Jian Kuang) has significant statistical expertise.

### Methodology

• retrospective

• diagnostic or prognostic study / observational

• performed at one institution
