# Supplementary material for: A new discriminant strategy combined with four TIRADS screening procedures increases ultrasound diagnostic accuracy—focusing on “wrong diagnostic” thyroid nodules
Source: Eur Radiol. 2022 Sep 28;33(2):784–96. doi: 10.1007/s00330-022-09126-2 (PMC9889473; doi:10.1007/s00330-022-09126-2)
Supplement: Supplementary file 1 — Supplementary file1 (DOCX 84.6 KB) [file 330_2022_9126_MOESM1_ESM.docx]

Supplementary Table1 Comparison of ultrasound lexicon of the four TIRADS

| Ultrasound Lexicon |  | ACR-TIRADS | Kwak-TIRADS | C-TIRADS | EU-TIRADS | Note |
| --- | --- | --- | --- | --- | --- | --- |
| Composition | Classification | Cystic or almost completely cystic, Spongiform, Mixed cystic and solid, Solid or almost completely solid | Solid, Mixed solid, Cyst | Solid,Predominately solid,Predominately cystic,Cystic,Spongiform | Solid, Mixed predominantly solid, Mixed predominantly cystic, Cystic, Spongiform | Accordant |
|  | Solid composition | Solid or almost completely solid | No clear definition | Entirely composed of solid tissue, without any cystic components | Composed almost entirely of soft tissue with <10% of liquid | Almost accordant except subtle difference between EU-TIRADS and C-TIRADS  The solid composition feature of nodules in our study cohort meets the definition of C-TIRADS |
|  | Spongiform | Composed predominantly (>50%) of small cystic spaces (regardless of echogenicity or other ultrasound features) | Not mentioned | Multiple tiny cystic spaces occupy the entire nodules without aggregated solid tissues | Tiny cystic spaces separated by thin septa (regardless of echogenicity or other ultrasound features) | Discordant, ACR-TIRADS≠C-TIRADS≠  EU-TIRAD; treated as cystic in Kwak TIRADS  According to definition of each TIRADS in our study |
| Echogenicity | Classification | Very hypoechoic, Hypoechoic, Hyperechoic or isoechoic,Anechoic | Hyper/iso-echogenicity,Hypo-echogenicity,Marked hypoechogenicity | Markedly hypoechoic, Hypoechoic, Isoechoic,Hyperechoic,Anechoic | Markedly hypoechoic,Mildly hypoechoic,Isoechoic,Hyperechoic | Accordant |
|  | Mixed echogenicity | Predominant echogenicity of the solid component | Predominant echogenicity of the solid component | Predominant echogenicity of the solid component | Exisitence of hypoechoicity in solid nodule or the solid part of cystic nodule both indicate mildly hypoechoic nodule | Discordant, EU-TIRADS≠other three TIRADS  Specific definition for EU-TIRADS in our study |
| Margin | Classification | Smooth,Ill-defined,Lobulated or irregular, Extra-thyroidal extension | Well-circumscribed,  Microlobulated,Irregular | Circumscribed,Irregular margin, Ill-defined, Extrathyroidal extension | Smooth,Ill-defined,Irregular margin | Accordant |
| Orientation | Classification | Taller than wide, Wider than tall | Wider than tall, Taller than wide | Taller than wide, Wider than tall | Oval or round shape, Taller than wide,Taller than long | Almost accordant |
|  | section to evaluate the nodule orientation | Transverse section | Transverse section | Transverse or longitudinal section | Transverse or longitudinal section | Discordant,ACR and Kwak-TIRADS≠  C-TIRADS and EU-TIRADS  Transverse section was mostly used in our center |
| Calcification | Classification | None or large comet-tail artifacts, Macrocalcifications, Peripheral calcifications,Punctate echogenic foci | Microcalcifications,Macrocalcifications,Rim calcifications,Comet-tail artifact | Microcalcifications,Comet-tail artifacts,Punctate echogenic foci of undermined significance,  Macrocalcifications,Peripheral calcifications,No echogenic foci | Microcalcification,Macrocalcification,Comet tail, Egg shell calcification | Almost accordant |
|  | Comet tail artifacts | Echogenic foci with V-shaped echoes>1mm deep to them | No clear definition | Punctate echogenic foci with a dense tapering trail of echoes in the rear | Reverberation artifacts within the cystic component | Accordant |

Supplementary Table 2 Comparison of construction and categories of the four TIRADS

|  | Model | Assessment of highly suspicious malignant features | Assessment of highly negative feature | Classfication | Assessment | Estimated  malignancy risk |
| --- | --- | --- | --- | --- | --- | --- |
| ACR-TIRADS | Score-based | Markedly hypoechoic, Taller than wide,Microcalcifications (Punctate echogenic foci) | None | TR1 | 0 points, Benign | ≤2% |
|  |  |  |  | TR2 | 2 points, Not suspicious | ≤2% |
|  |  |  |  | TR3 | 3 points,Mildly suspicious | ＜5% |
|  |  |  |  | TR4 | 4-6 points, Moderately suspicious | 5%-20% |
|  |  |  |  | TR5 | 7 or more points, Highly suspicious | ＞20% |
| Kwak-TIRADS | Counting-based | Solid, Hypoechoic or Markedly hypoechoic,Microlobulated or irregular,Microcalcifications, Taller than wide | None | 1 | Negative | 0% |
|  |  |  |  | 2 | Benign | 0% |
|  |  |  |  | 3 | Probably benign | 2.0-2.8% |
|  |  |  |  | 4a | Low suspicion for malignancy  (1 suspicious ultrasound feature) | 3.6-12.7% |
|  |  |  |  | 4b | Intermediate suspicion for malignancy  (2 suspicious ultrasound features) | 6.8-37.8% |
|  |  |  |  | 4c | Moderate concern but not classic for malignancy  (3 or 4 suspicious ultrasound features) | 21-91.9% |
|  |  |  |  | 5 | Highly suggestive of malignancy  (5 suspicious ultrasound features) | >95% |
| C-TIRADS | Counting-based | Taller than wide,Solid, Markedly hypoechoic,Microcalcifications,  Ill-defined/irregular margin or extrathyroidal extension | Comet tail artifacts | CTR1 | No nodules | 0% |
|  |  |  |  | CTR2 | -1 Point | 0% |
|  |  |  |  | CTR3 | 0 Points | ≤2% |
|  |  |  |  | CTR4a | 1 Points | 2-10% |
|  |  |  |  | CTR4b | 2 Points | 10-50% |
|  |  |  |  | CTR4c | 3-4 Points | 50-90% |
|  |  |  |  | CTR5 | 5 Points | >90% |
|  |  |  |  | CTR6 | Proved Malignant | - |
| EU-TIRADS | Pattern-based | Irregular shape, Irregular margins, Microcalcifications,  Marked hypoechogenicity | None | 1 | Normal  No nodules | None |
|  |  |  |  | 2 | Benign  Pure cyst,Entirely spongiform | 0% |
|  |  |  |  | 3 | Low risk  Ovoid,smooth isoechoic/hyperechoic  No features of high suspicion | 2-4% |
|  |  |  |  | 4 | Intermediate risk  Ovoid,smooth,mildly hypoechoic  No features of high suspicion | 6-17% |
|  |  |  |  | 5 | High risk  At least 1 of the five features of high suspicion  mentioned | 26-87% |

Supplementary Table 3 Demographics and ultrasound characteristics of the thyroid nodules among patients

|  | | Total | Benign | Malignant | *P* |
| --- | --- | --- | --- | --- | --- |
| No. of nodules | | 795 | 461 | 334 | - |
| Mean age (y) | | 43.49±12.52 | 43.89±12.88 | 42.82±11.89 | 0.349 |
| Gender | |  |  |  | 0.072 |
|  | Male | 173(21.8%) | 90(19.5%) | 83(24.9%) |  |
|  | Female | 622(78.2%) | 371(80.5%) | 251(75.1%) |  |
| Blood test | |  |  |  |  |
|  | TSH (IU/mL) | 1.00(0.62,1.40) | 1.05(0.63,1.47) | 0.96(0.53,1.27) | 0.146 |
|  | FT3 (pmol/L) | 5.32±0.88 | 5.37±0.92 | 5.27±0.86 | 0.353 |
|  | FT4 (pmol/L) | 11.77±2.70 | 11.61±2.34 | 11.92±2.98 | 0.369 |
|  | TPOAb(U/mL) | 13.04(9.54, 18.47) | 12.40(9.57,17.26) | 13.83(9.30,19.30) | 0.303 |
|  | TGAb(U/mL) | 13.00(10.00, 28.96) | 12.28(10.00, 19.03) | 13.30(10.00,45.31) | 0.390 |
|  | TRAb(IU/L) | 0.30(0.30, 0.44) | 0.30(0.30,0.36) | 0.30(0.30,0.50) | 0.418 |
|  | TG(ng/mL) | 14.00(7.07, 30.62) | 15.34(7.46,32.23) | 13.01(5.86,25.97) | 0.112 |
|  | hCT(ng/L) | 1.00(0.50, 2.30) | 1.00(0.50,2.03) | 1.38(0.50,2.68) | 0.324 |
| Ultrasound features | |  |  |  |  |
|  | Size(cm) | 1.60(1.00, 2.80) | 2.40(1.50, 3.20) | 1.00(0.80, 1.50) | ＜0.001 |
|  | Size＜1cm | 191(24.0%) | 47(10.2%) | 144(43.1%) | ＜0.001 |
|  | Solid composition | 638(80.3%) | 313(67.9%) | 325(97.3%) | ＜0.001 |
|  | Hypoechogenicity or marked  Hypoechogenicity | 409(51.4%) | 95(20.6%) | 314(94.0%) | ＜0.001 |
|  | Taller than wide | 131(16.5%) | 15(3.2%) | 116(34.7%) | ＜0.001 |
|  | Lobulated or irregular | 335(42.1%) | 60(13.0%) | 275(82.3%) | ＜0.001 |
|  | Ill-defined margin | 378(47.5%) | 88(19.1%) | 299(89.5%) | ＜0.001 |
|  | Extrathyroidal extension | 11(1.4%) | 0(0%) | 11(3.3%) | ＜0.001 |
|  | Microcalcifications | 219(27.5%) | 28(6.1%) | 191(57.2%) | ＜0.001 |
|  | Macrocalcifications | 110(13.4%) | 69(14.9%) | 29(8.7%) | 0.008 |
|  | Peripheral calcifications | 7(0.9%) | 6(1.3%) | 1(0.3%) | 0.249 |

hCT, human calcitonin

Supplementary Table 4 Diagnostic performance of the four TIRADS

|  | Cut off | Sensitivity  (%) | Specificity (%) | PPV (%) | NPV (%) | AUC (95% CI) |
| --- | --- | --- | --- | --- | --- | --- |
| ACR-TIRADS | TR5 | 81.4 | 91.1 | 86.9 | 87.1 | 0.863 (0.837-0.886) |
| Kawk-TIRADS | 4c | 88.3 | 88.5 | 84.8 | 91.3 | 0.884 (0.860-0.906) |
| C-TIRADS | CTR4b | 91.6 | 80.9 | 77.7 | 93.0 | 0.863 (0.837-0.886) |
| EU-TIRADS | 5 | 91.0 | 84.0 | 80.4 | 92.8 | 0.875 (0.850-0.897) |

PPV: Positive predictive value; NPV: Negative predictive value; AUC, Area under the curve; CI: Confidence interval

Supplementary Table 5 Ultrasound features for the nodules subgroups partially inconsistently with diagnosis screened by the four TIRADS

|  | | | Total | Benign | Malignant |  |
| --- | --- | --- | --- | --- | --- | --- |
| No. of nodules | | | 95 | 55 | 40 |  |
| Size(cm) | | | 1.30(0.80, 2.40) | 2.10(0.90, 3.00) | 1.00(0.63, 1.30) | |
| Composition | | |  |  |  |  |
|  | Solid | | 84(88.4%) | 47(85.5%) | 37(92.5%) |  |
|  | Mixed cystic and solid | | 11(11.6%) | 8(14.5%) | 3(7.7%) |  |
| Echogenicity | | |  |  |  |  |
|  | Hyper-/Iso-echogenicity | | 43(40%) | 39(70.9%) | 4(10%) |  |
|  | Hypoechogenicity | | 49(56.8%) | 15(27.3%) | 34(85%) |  |
|  | Marked  Hypoechogenicity | | 3(3.2%) | 1(1.8%) | 2(5%) |  |
| Orientation | | |  |  |  |  |
|  | Wider than tall | | 94(98.9%) | 55(100%) | 39(97.5%) |  |
|  | Taller than wide | | 1(1.1%) | 0(0%) | 1(2.5%) |  |
| Margin | | |  |  |  |  |
|  | Well circumscribed | | 40(42.1%) | 31(56.4%) | 9(22.5%) |  |
|  | Lobulated or irregular | | 55(57.9%) | 24(43.6%) | 30(77.5%) |  |
| Calcification | |  |  |  |  |  |
|  | No calcifications | | 68(71.6%) | 36(65.5%) | 32(80.0%) |  |
|  | Macrocalcifications | | 19(20.0%) | 13(23.6%) | 6(15.0%) |  |
|  | Microcalcifications | | 8(8.4%) | 6(10.9%) | 2(5.0%) |  |

Supplementary Table 6 Diagnostic performance of the SP+DS method and four TIRADS in subgroups of initial Bethesda 3 and 4 nodules with pathologically confirmed diagnosis

|  |  | ACR-TIRADS | Kwak-TIRADS | EU-TIRADS | C-TIRADS | SP+DS |
| --- | --- | --- | --- | --- | --- | --- |
| Pathological benign | 14 | 7(50.0%) | 6(42.9%) | 3(21.4%) | 4(28.6%) | 6(42.9%) |
| Pathological malignance | 17 | 10(58.8%) | 13(76.5%) | 15(88.2%) | 16(94.1%) | 14(82.4%) |
| Total | 31 | 17(54.8%) | 19(61.3%) | 18(58.1%) | 20(64.5%) | 20(64.5%) |

SP+DS: The evaluation method consists of the four TIRADS screening procedures with partially inconsistently diagnosed nodules judged by discriminant strategy
